# Moment-to-Moment Interplay Among Stress Appraisals and Emotion Regulation Flexibility in Daily Life

**DOI:** 10.1007/s42761-022-00122-9

**Published:** 2022-06-06

**Authors:** Angela Socastro, Jonas Everaert, Teresa Boemo, Ivan Blanco, Raquel Rodríguez-Carvajal, Alvaro Sanchez-Lopez

**Affiliations:** 1 Department of Personality, Evaluation and Psychological Treatment, Faculty of Psychology, Complutense University of Madrid, Somosaguas Campus, 28223 Madrid, Spain; 2 Department of Medical and Clinical Psychology, Tilburg University, Tilburg, The Netherlands; 3 Research Group of Quantitative Psychology and Individual Differences, KU Leuven, Leuven, Belgium; 4 Department of Biological and Health Psychology, Autonoma University of Madrid, Madrid, Spain

**Keywords:** Emotion regulation flexibility, Rumination, Reappraisal, Active coping, Avoidance, Stress controllability

## Abstract

Flexible use of emotion regulation (ER) strategies in daily life is theorized to depend on appraisals of occurring stressful events. Yet, to date, little is known about (a) how appraisals of the current situation modulate the use of ER strategies in daily life and (b) how individual differences in affective symptoms impact these relations among appraisals and ER strategy use. This study attempted to address these two limitations using a 5-day experience sampling protocol, with three surveys administered per day in a sample of 97 participants. Each survey measured momentary appraisals of stress intensity and controllability as well as ER strategy use (i.e., rumination, reappraisal, avoidance, and active coping). Results showed that, in situations of low-stress intensity, higher stress controllability was related to greater use of reappraisal and rumination. In situations of high-stress intensity, higher controllability was related to reduced use of rumination. This pattern of flexible use of ER strategies depending on momentary stress appraisals was found for both rumination and avoidance and occurred specifically in individuals reporting lower levels of depression and/or anxiety levels. These findings provide new insight into how flexible use of ER strategies in daily life is modulated by interactions between stress intensity and controllability appraisals at varying levels of affective symptoms.

**Supplementary Information:**

The online version contains supplementary material available at 10.1007/s42761-022-00122-9.

Emotion regulation is integral to multiple areas of psychosocial functioning (John & Gross, 2004; Nezlek & Kuppens, 2008) and refers to processes to modulate the intensity, frequency, and duration of emotional responses (Gross, 2014). Specifically, the ability to effectively regulate negative emotions in stressful experiences is critical to emotional well-being (Myruski et al., 2018). Research in this field has traditionally considered certain emotion regulation (ER) strategies as maladaptive (e.g., rumination, avoidance), whereas other ER strategies (e.g., reappraisal, problem-solving) were viewed as adaptive in downregulating emotional distress (Aldao et al., 2010). However, recent perspectives propose that effective ER depends on one’s ability to flexibly implement different ER strategies depending on contextual demands (Aldao et al., 2015). Thus, appraisals of the current situation are expected to shape which ER strategies are selected and implemented to regulate an emotional experience based on the characteristics of a particular situation (Bonanno & Burton, 2013; Everaert et al., 2021; Mehu & Scherer, 2015). Current perspectives propose that the appraisal processes informing such flexible use of ER strategies involve weighing different aspects of the stressful situation (i.e., stress intensity) and its coping potential (i.e., stress controllability, e.g., Goodman et al., 2021; Haines et al., 2016).

Empirical research has shown that higher perceived stress intensity is associated with greater overall use of ER strategies (e.g., Dixon-Gordon et al., 2015), and that such stress intensity appraisals may further influence which ER strategies are used. In high-intensity situations, people may prefer using distraction or mental avoidance (i.e., trying to move mental focus away from unwanted internal thoughts, physical sensations, and emotions) instead of reappraisal (i.e., changing the individual’s perspective or interpretation of the stressful situation in a more benign way), whereas reappraisal may be preferred in low-intensity situations (Sheppes et al., 2011, 2014). Stress controllability appraisals have also been found to influence the preferential use of different forms of ER strategies. Studies of individuals experiencing high levels of stress suggest that reappraisal is used more frequently in situations that are appraised as uncontrollable (Troy et al., 2013). Active coping (i.e., problem-solving strategies aimed to modify the situation) would be preferred in stressful situations that are perceived as controllable (Troy, 2015). As for rumination (i.e., repetitive thinking about one’s negative emotions and experiences, their causes, and consequences), some studies suggest that it can be used to cope with controllable situations that allow for efficient affective processing (i.e., characterized by low-stress intensity; see Nolen-Hoeksema et al., 2008).

Other studies have considered the relation between controllability appraisals and the momentary use of ER strategies during daily life functioning. In an 8-day daily-diary study, David and Suls (1999) found that higher perceived stress controllability was related to higher use of active coping and lower use of avoidance. In a study using a 7-day experience sampling method (ESM), Haines et al. (2016) found that individuals reporting lower levels of depression and anxiety symptoms used more reappraisal in uncontrollable situations as compared to individuals reporting higher symptom levels. Finally, an ESM study in clinical and healthy individuals by Kircanski et al. (2015) showed that lower momentary levels of perceived controllability predicted a higher use of rumination.

These previous ESM studies have considered the influence of momentary stress controllability on ER strategy use as a single predictor without considering stress intensity. However, based on theoretical (e.g., Bonanno & Burton, 2013) and experimental studies (e.g., Troy et al., 2013), it is plausible that controllability appraisals are related to the use of ER strategies depending on the level of stress intensity in each situation. Addressing this limitation, the current study tested the hypothesis that momentary controllability appraisals are related to subsequent differential use of reappraisal, active coping, avoidance, and rumination strategies. These relations between controllability appraisals and ER strategy use were hypothesized to be moderated by momentary stress intensity appraisals as it follows.

First, it was expected that appraisals of lower stress controllability would be related to higher use of reappraisal, particularly in conditions of low-stress intensity (Sheppes et al., 2009, Haines et al., 2016; Troy et al., 2013). No specific relations were expected for conditions marked by high-stress intensity. This is because prior research suggests that reappraisal is a strategy that is particularly used in low-intensity conditions (Sheppes et al., 2011, 2014).

Second, in line with theoretical predictions (Troy, 2015) and empirical evidence (David & Suls, 1999), it was expected that higher momentary controllability appraisals would be related to higher use of active coping, irrespective of momentary stress intensity levels. In contrast, avoidance use has been related to both higher appraisals of stress intensity (Sheppes et al., 2011, 2014) and lower appraisals of controllability (David & Suls, 1999). Thus, it was hypothesized that, in situations of high intensity, lower controllability appraisals would be related to higher use of avoidance. No specific predictions were made for avoidance use in conditions of low intensity.

Finally, it has been proposed that momentary rumination can assist problem-solving in specific conditions, allowing for efficient affective processing (i.e., high controllability at low-stress intensity; Nolen-Hoeksema et al., 2008), and empirical ESM evidence has supported a general negative association between momentary stress controllability and rumination use (Kircanski et al., 2015). Based on these findings, it was hypothesized that lower controllability appraisals would be related to higher rumination use in general, but that higher controllability appraisals would be related to greater rumination use in situations of high-stress intensity.

The above predicted appraisal-ER strategy use connections are expected to reflect how ER flexibility is expressed in daily life, as evidenced by within-individual variance in the use of each specific ER strategy, depending on momentary stress appraisals (see also Goodman et al., 2021). ER flexibility is thought to be a central marker of adaptive emotional functioning (Aldao et al., 2015) and is impaired in conditions of high affective symptomatology such as elevated depression or anxiety levels (Bonanno & Burton, 2013; Cheng et al., 2014; Haines et al., 2016). In the present study, it was further examined whether the hypothesized flexible patterns of ER strategy use in stressful contexts are modulated by individual differences in depression and/or anxiety levels. Previous research has shown that individuals reporting high levels of affective symptoms are characterized by both rigid negatively biased stress appraisals (e.g., Everaert et al., 2020; see also Scherer, 2021) and inflexible use of ER strategies across situations irrespective of those appraisals (e.g., Goodman et al., 2021). Therefore, it was tested whether the above predicted appraisal-ER strategy use relations would occur at lower depression and anxiety levels (see Fig. 1). In contrast, at higher affective (depression/anxiety) symptom levels, more rigid patterns of stress appraisals and ER strategy use were expected, with ER strategies being used independent of momentary stress appraisals.

**Fig. 1:**
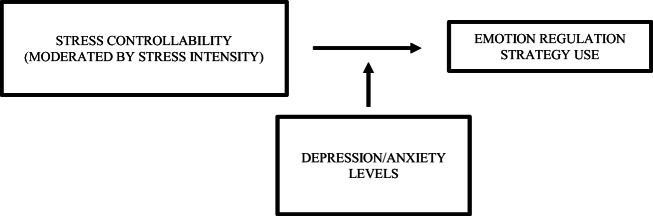
Graphic depiction of the hypothesized model

## Method

### Participants

A sample of 105 undergraduates (*M*_age_=20.13 years, SD*=*2.43; 87% female) from the Autonomous University of Madrid and the Complutense University of Madrid enrolled in the study. Participants received course credits for their participation. The sample size was determined following current recommendations of required level 2 and level 1 sample sizes in ESM studies (Gabriel et al., 2019; *n*=83 in level 2 and *n*=835 in level 1 as minimum adequate sizes). In this study, the final sample was *n*=97 in level 2 and *n*=1, 302 in level 1 (see below). The study was approved by the Faculty Ethical Committee of Complutense University of Madrid (Protocol Code Ref. 2019/20-028) and complied with Helsinki Declaration’s Ethics Standards.

### Procedure

After providing informed consent, participants met with an experimenter who installed a custom-built ESM app on their phones (Android or IOS operating systems). Participants were then trained on how to complete the ESM protocol on their phones during the following 5 days. The session concluded with a set of questionnaires measuring anxiety and depression symptoms (see below) via Qualtrics software (Qualtrics, Provo, UT).

Next, participants completed the main experimental ESM protocol during the following 5 days. The delivery plan of the ESM paradigm was modeled after prior ESM research studying temporal changes in ER strategy use (Connolly & Alloy, 2017; Heiy & Cheavens, 2014). The ESM paradigm comprised online assessments for 5 days, during which participants were prompted 3 times per day to complete an ESM survey. Notifications were sent pseudo-randomly within pre-specified 4-h periods between 9 a.m. and 9 p.m. (9 a.m.–1 p.m., 1 p.m.–5 p.m., and 5 p.m.–9 p.m.) to obtain a representative sample of experiences during the day. Participants had 1 h to complete each ESM survey following the notification (see below for details). After completing the 5-day ESM protocol, participants completed a brief questionnaire assessing the perceived usability of the ESM app and were fully debriefed.

## Materials^1^

### Baseline pre-ESM Assessments of Affective Symptoms

#### Depressive Symptoms

The Center for Epidemiologic Studies - Depression Scale 8 item version (CES-D 8; Radloff, 1977) was used to evaluate depressive symptom levels. The CES-D 8 is an 8-item screening test that measures depressive symptom severity levels referring to the last week (e.g., “you felt sad”, “you felt everything you did was an effort”). Items are rated on a 4-point Likert Scale, from 0 (rarely or none of the time) to 3 (most or all of the time). The total score ranges from 0 to 24. The cutoff point is 9 (Beller et al., 2020), with scores up or equal to 9 indicating the possible presence of clinical depressive symptoms. In the current sample, 34% of participants were above the CES-D cutoff criterion for clinical depression. This measure has good reliability and validity both in general and in depressed samples (Karim et al., 2015; Van de Velde et al., 2009). In the current study, the internal consistency of the CES-D 8 was 0.92.

#### Anxiety Symptoms

To assess anxiety symptoms, we used the generalized anxiety disorder scale (GAD-7; Spitzer et al., 2006). The GAD-7 is a 7-item screening test that measures anxiety symptom severity levels referring to the last 2 weeks (e.g., “feeling nervous, anxious or on edge”, “not being able to stop or control worrying”). Items are rated on a 4-point Likert scale, from 0 (not at all) to 3 (nearly every day). The total score ranges from 0 to 21. The cutoff point of this screening test is 10, with scores above 10 indicating the possible existence of clinical symptoms of anxiety (Spitzer et al., 2006). In the current study, 11.3% of participants in the sample were above the GAD cutoff criterion for clinical anxiety symptomatology. This measure has shown good reliability and validity in both general and anxious samples (Löwe et al., 2008; Ruiz et al., 2011). In the current study, the internal consistency of the GAD-7 was 0.92.

### ESM Measures

#### Stress Appraisals

Perceived stress intensity and stress control were measured at each ESM survey. Two questions measured these appraisals with respect to the most stressful situation since the last survey (see also Haines et al., 2016). Perceived stress intensity was measured with the item “Think about the most important event since the last survey. To what extent has that situation been stressful to you?” rated in a scale from 0 (not all) to 10 (very much): Perceived stress control was measured using the item “To what extent were you in control of that situation that has happened to you since the last survey?” using a rating scale from 0 (not all) to 10 (very much).

#### Main ER Strategies

This study focused on the use of four ER strategies, namely reappraisal, active coping, avoidance, and rumination. In every ESM survey, participants were asked about the extent they used each ER strategy with respect to the most stressful situation encountered since the last survey. All measures of ER strategy use were rated on a scale from 0 (not all) to 10 (very much).

Reappraisal was assessed using the item “… have you looked at things from a different perspective?” (see also Haines et al., 2016). Active coping was measured using the item “… have you tried to change something about the situation?” (Haines et al., 2016): Avoidance was measured with the item “… have you tried to avoid thinking about your feelings and problems?” (Aldao et al., 2010): Rumination was measured using the item “… have you been dwelling on your feelings and problems?”, which is in line with prior work (Kircanski et al., 2015).

#### Other ER Strategies

As with the four main ER strategies under study for which specific hypotheses were formulated, participants answered in every ESM survey to what extent they had used four further ER strategies. Worry: Following previous ESM research (Kircanski et al., 2015), worry use in response to ongoing stressful experiences was assessed with the item “… have you been worrying about things that could happen?”. Mental change: A further item used in previous ESM research monitoring different forms of momentary cognitive reappraisal use in response to stress was used, phrased as follows “…have you changed the way you were thinking about the situation?” (Haines et al., 2016). External distraction: This ER strategy was measured with the item: “… have you tried to distract yourself from what was going on?”. Future Planning: It was phrased in accordance with problem-solving models (D’Zurilla et al., 2004) considering future planning as an alternative form of problem-solving coping, as follows: “… have you thought about what you could do to solve similar upcoming situations in the future?” Despite that no specific hypotheses were formulated for this further set of ER strategies, the relevance of these strategies to furthering models of ER flexibility remains apparent. As such, exploratory analyses were conducted considering their association with ongoing stress controllability appraisals, modulated by stress intensity appraisals and individual differences in depression and anxiety levels, similar to the analyses for the main ER strategies under study. The full set of these analyses is further provided in Supplement 2.

### Data Cleaning

Attrition in this study was low. Three participants did not complete the study protocol. Compliance rates were examined, and participants were considered only if they responded to at least the 75% (11 out of 15) of prompts. Five participants did not meet this criterion and were excluded. On average, participants completed 13 out of 15 prompts (SD=0.30; range: 9 to 15). The final sample comprised of 97 participants (mean age: 20.13 years, 87% female). There were no significant differences between completers and non-completers with respect to depression scores, *t*(100)= 0.531, *p=*0.590, or anxiety levels, *t*(100)= 1.961, *p=*0.114. Data were also considered in terms of the time elapsed between notification prompts and actual times of response. On average, participants took 19 min (SD= 8.67, range: 2.13–54.07 min) to complete ESM surveys since notification. Overall, the protocol reflected a high degree of compliance both in terms of completed surveys and in time elapsed to response to prompts.

### Statistical Analyses

Multilevel modeling was used given the nested data structure, with surveys (*t*: 1–15) nested within individuals (*j*: 1–97). The maximum likelihood method was used to estimate the models. The analyses were performed using the *lme* function of the *nlme* package for R (Pinheiro et al., 2021). At level 1, predictors in each model (i.e., stress control and stress intensity) were person-mean centered, thus removing between-person differences. At level 2 (j), predictors (i.e., depressive and anxiety symptom levels) were grand-mean centered (Aguinis et al., 2013; Ohly et al., 2010). Assumptions of normality and homoscedasticity were tested and within- and between-individual outlier analyses were performed. No data transformations were required. Unconditional (empty) models were first fitted for each variable to estimate the mean and variance at each level. Intraclass correlations (ICC) were computed from these models to decompose total variance into between- and within-person variance components.

Next, we constructed a series of multilevel models examining whether ER strategy use at time ‘*t*’ was predicted by stress intensity and control appraisal levels as well as their interaction. A two-level model was fitted for each ER strategy variable with random intercepts and slopes (Aguinis et al., 2013; Bolger & Laurenceau, 2013). Both stress intensity and control as well as their interaction term at ‘t’ were added as predictors of ER strategy level at ‘t’. Depression and anxiety levels were further modeled to test whether individual differences in each type of affective symptoms impacted the relation between stress appraisals and subsequent use of each of the ER strategies (i.e., Fig. 1). Thus, at level 2, we modeled the random intercepts and slopes of ER strategies as a function of individual differences in each measure of affective symptoms (i.e., depression and anxiety symptom levels; grand-mean centered CESD and GAD scores). The models are specified below and fitted separately for each level 2 predictor.

$$ {\displaystyle \begin{array}{c}\mathrm{ER}\ {\mathrm{strategies}}_{\mathrm{tj}}={\mathrm{p}}_{\mathrm{oj}}+{\mathrm{p}}_{1\mathrm{j}}\left({\mathrm{stress}\ \mathrm{control}}_{\mathrm{tj}}\right)+{\mathrm{p}}_{2\mathrm{j}}\left({\mathrm{stress}\ \mathrm{intensity}}_{\mathrm{tj}}\right)+{\mathrm{p}}_{3\mathrm{j}}\left({\mathrm{stress}\ \mathrm{intensity}}_{\mathrm{tj}}\right)\left({\mathrm{stress}\ \mathrm{control}}_{\mathrm{tj}}\right)+{\mathrm{e}}_{\mathrm{tj}}\\ {}{\mathrm{p}}_{0\mathrm{j}}={\mathrm{b}}_{00}+{\mathrm{b}}_{01}\left({\mathrm{depression}}_{\mathrm{j}}/{\mathrm{anxiety}}_{\mathrm{j}}\right)+{\mathrm{r}}_{0\mathrm{j}}\\ {}{\mathrm{p}}_{1\mathrm{j}}={\mathrm{b}}_{10}+{\mathrm{b}}_{11}\left({\mathrm{depression}}_{\mathrm{j}}/{\mathrm{anxiety}}_{\mathrm{j}}\right)+{\mathrm{r}}_{1\mathrm{j}}\\ {}{\mathrm{p}}_{2\mathrm{j}}={\mathrm{b}}_{20}+{\mathrm{b}}_{21}\left({\mathrm{depression}}_{\mathrm{j}}/{\mathrm{anxiety}}_{\mathrm{j}}\right)+{\mathrm{r}}_{2\mathrm{j}}\\ {}{\mathrm{p}}_{3\mathrm{j}}={\mathrm{b}}_{30}+{\mathrm{b}}_{31}\left({\mathrm{depression}}_{\mathrm{j}}/{\mathrm{anxiety}}_{\mathrm{j}}\right)+{\mathrm{r}}_{3\mathrm{j}}\end{array}} $$

When stress intensity × stress control interactions were statistically significant, slopes of the corresponding ER strategy were regressed on stress control appraisal levels, being computed separately for occasions with average stress intensity levels, 1 SD above, and 1 SD below average levels (i.e., high and low-stress intensity occasions, respectively). Furthermore, exploratory models considering stress control as the moderator of the relationship between stress intensity and ER strategy use were also conducted to fully disentangle supported 2-way interactions. Thus, slopes of the corresponding ER strategy were further regressed on stress intensity appraisal levels, being computed separately for occasions with average stress controllability levels, 1 SD above, and 1 SD below average levels. When three-way interactions considering 2-level depression and/or anxiety as moderators were statistically significant, the described slopes were computed separately for individuals with average depression and/or anxiety levels, 1 SD above and 1 SD below average levels.

## Results^2^

### Preliminary analyses

Means, standard deviations, and intraclass correlation coefficients (ICCs) were estimated for stress appraisal and ER strategies variables using empty-intercept models. Table 1 shows the mean level with the 95% CI, the standard deviation of the within- and between-person levels, the ICC, and the percentage of the total variability between- and within-subject for all the variables. The ICCs suggest that there was a large variation at the within-person level in all cases. Thus, individuals showed clear fluctuations in their stress appraisals levels and their use of each ER strategy across different daily situations during the ESM protocol.

**Table 1:** Mean level, SDs of the within- and between-person levels, ICC and percentage of the total variability between and within-subject for Stress Appraisals and use of Rumination, Reappraisal, Avoidance and Active Coping

Intercept-only model	Mean level Estimate 95% CI (Lower-Upper)	SD Between subject	SD Within-subject	ICC	% Between subjects variance	% Within-subject variance
Stress Intensity	3.74 (3.44-4.03)	1.30	2.57	0.20	20.44%	79.55%
Stress Control	5.84 (5.53-6.16)	1.45	2.20	0.30	30.35%	69.64%
Rumination	4.58 (4.18-4.97)	1.89	2.28	0.40	40.75%	59,24%
Reappraisal	3.99 (3.68-4.30)	1.42	2.17	0.30	30.10%	69.89%
Avoidance	3.58 (3.26-3.90)	1.47	2.13	0.32	32.29%	67.70%
Active Coping	3.85 (3.53-4.17)	1.46	2.23	0.30	30.04%	69.95%

Table 2 shows the effect of individual differences in depression and anxiety levels on momentary levels of stress intensity and controllability appraisals. This served as a preliminary step to establish the extent to which individual differences in affective symptoms impact the stress appraisal levels across the ESM protocol. The results indicated that higher levels of depression and anxiety were both related to higher appraisals of stress intensity and lower appraisals of stress control across the 5 days.

**Table 2:** Results on the relations between depression and anxiety levels and stress appraisals across the study

Model	Fixed Effects	Estimate.	*SE*	*t*	*p*
Stress Intensity at t	Intercept	3.740	0.138	27.193	<.0.001
	Depression	0.146	0.037	3.935	<.0.001
	Intercept	3.738	0.116	32.098	<0.001
	Anxiety	0.237	0.031	7.740	<0.001
Stress Control at t	Intercept	5.847	0.134	43.501	<0.001
	Depression	-0.215	0.034	-6.374	<0.001
	Intercept	5.838	0.140	41.802	<0.001
	Anxiety	-0.200	0.039	-5.104	<0.001

### Main Results

Table 3 provides statistics for each 2-level model testing the role of stress intensity, stress control, affective symptoms (i.e., depression or anxiety levels), and their interactions in accounting for the momentary use of each ER strategy. Table 4 shows the significant simple slopes for each model when 2-way or 3-way interactions were statistically supported.

**Table 3:** Results from the main models

	Fixed effects				
Model	Effect	Estimate.	*SE*	*t*	*p*
Reappraisal at t	Intercept	3.950	0.159	24.780	<0.001
	Stress Intensity at t	0.131	0.030	4.261	<0.001
	Stress Control at t	0.058	0.029	1.969	0.049
	Depression at t	-0.032	0.040	-0.813	0.418
	Stress Intensity x Stress Control	-0.028	0.009	-2.915	0.003
	Stress Intensity x Depression	-0.012	0.008	-1.535	0.125
	Stress Control x Depression	0.013	0.008	1.591	0.111
	Stress Intensity x Stress Control x Depression	0.001	0.002	0.250	0.802
	Anxiety at t	0.013	0.041	0.326	.745
	Stress Intensity x Stress Control	-0.028	0.009	-2.964	0.003
	Stress Intensity x Anxiety	-0.015	0.008	-1.817	0.069
	Stress Control x Anxiety	0.021	0.008	2.535	0.011
	Stress Intensity x Stress Control x Anxiety	0.004	0.003	1.494	0.135
Active Coping at t	Intercept	3.846	0.162	23.613	<0.001
	Stress Intensity at t	0.232	0.034	6.789	<0.001
	Stress Control at t	0.004	.029	0.140	0.888
	Depression at t	0.010	0.041	0.264	0.792
	Stress Intensity x Stress Control	-0.005	0.009	-0.591	0.554
	Stress Intensity x Depression	-0.012	0.008	-1.383	0.166
	Stress Control x Depression	0.010	0.008	1.233	0.217
	Stress Intensity x Stress Control x Depression	-0.002	0.003	-0.773	0.439
	Anxiety at t	0.076	0.041	1.874	0.063
	Stress Intensity x Stress Control	-0.005	0.009	-0.592	0.553
	Stress Intensity x Anxiety	0.004	0.009	0.508	0.611
	Stress Control x Anxiety	0.017	0.008	2.099	0.036
	Stress Intensity x Stress Control x Anxiety	0.005	0.003	1.641	0.101
Avoidance at t	Intercept	3.605	0.158	22.772	<0.001
	Stress Intensity at t	0.048	0.028	1.707	0.088
	Stress Control at t	.066	0.035	1.876	0.060
	Depression at t	0.102	0.039	2.583	0.011
	Stress Intensity x Stress Control	0.012	0.009	1.300	0.193
	Stress Intensity x Depression	-0.013	0.007	-1.799	0.072
	Stress Control x Depression	0.001	0.009	0.049	0.960
	Stress Intensity x Stress Control x Depression	0.007	0.002	2.383	0.017
	Anxiety at t	0.104	0.040	2.552	0.012
	Stress Intensity x Stress Control	0.013	0.009	1.390	0.164
	Stress Intensity x Anxiety	-0.017	0.007	-2.246	0.024
	Stress Control x Anxiety	-0.006	0.009	-0.662	0.507
	Stress Intensity x Stress Control x Anxiety	0.008	0.003	2.671	0.007
Rumination at t	Intercept	4.530	0.186	24.279	<0.001
	Stress Intensity at t	0.247	0.031	7.875	<0.001
	Stress Control at t	0.010	0.034	0.297	0.766
	Depression at t	0.215	0.469	4.583	<0.001
	Stress Intensity x Stress Control	-0.032	0.010	-3.264	0.001
	Stress Intensity x Depression	0.010	0.008	1.219	0.227
	Stress Control x Depression	-0.013	0.009	-1.476	0.140
	Stress Intensity x Stress Control x Depression	0.005	0.003	1.909	0.056
	Anxiety at t	0.287	0.043	6.536	<0.001
	Stress Intensity x Stress Control	-0.031	0.010	-3.109	0.001
	Stress Intensity x Anxiety	0.06	0.008	0.770	0.440
	Stress Control x Anxiety	0.002	0.009	-0.233	0.815
	Stress Intensity x Stress Control x Anxiety	0.003	0.003	0.959	0.337

**Table 4:** Summary of simple slopes analyses

Interaction SIxSC ➔ Reappraisal					
Predictor: SC					
SI	Test Estimate	SE	T values	df	p
Low	0.122	0.039	3.287	1202	0.001**
Mean	0.058	0.030	1.960	1202	0.050
High	-0.013	0.037	-0.346	1202	0.729
Predictor: SI					
SC	Test Estimate	SE	T values	Df	p
Low	0.019	0.036	5.221	1202	1.163e-07***
Mean	0.132	0.031	4.219	1202	2.638e-05***
High	0.071	0.039	1.829	1202	0.067
Interaction SIxSCxDepression ➔ Avoidance					
Individuals with lower depression levels					
Predictor: SC					
SI	Test Estimate	SE	T values	Df	p
Low	0.103	0.067	1.542	1199	0.123
Mean	0.065	0.052	1.258	1199	0.209
High	0.027	0.061	0.439	1199	0.661
Predictor: SI					
SC	Test Estimate	SE	T values	df	p
Low	0.135	0.050	2.758	1199	0.001**
Mean	0.102	0.039	2.582	1199	0.009**
High	0.070	0.054	1.312	1199	0.190
Individuals with higher depression levels					
Predictor: SC					
SI	Test Estimate	SE	T values	df	p
Low	-0.033	0.068	-0.486	1199	0.627
Mean	0.069	0.053	1.293	1199	0.196
High	0.170	0.064	1.682	1199	0.007**
Predictor: SI					
SC	Test Estimate	SE	T values	df	p
Low	-0.093	0.053	-1.769	1199	0.078.
Mean	-0.006	0.043	-0.140	1199	0.889
High	0.080	0.069	1.463	1199	0.145
Interaction SIxSCxAnxiety ➔ Avoidance					
Individuals with lower anxiety levels					
Predictor: SC					
SI	Test Estimate	SE	T values	df	p
Low	0.137	0.064	2.130	1199	0.033*
Mean	0.088	0.049	1.787	1199	0.074.
High	0.040	0.060	0.663	1199	0.506
Predictor: SI					
SC	Test Estimate	SE	T values	df	p
Low	0.159	0.051	3.135	1199	0.002**
Mean	0.117	0.040	2.921	1199	0.004**
High	0.076	0.053	1.433	1199	0.152
Individuals with higher anxiety levels					
Predictor: SC					
SI	Test Estimate	SE	T values	df	p
Low	-0.077	0.070	-1.110	1199	0.267
Mean	0.039	0.053	0.734	1199	0.463
High	0.155	0.063	2.444	1199	0.015*
Predictor: SI					
SC	Test Estimate	SE	T values	df	p
Low	-0.119	0.054	-2.180	1199	0.029*
Mean	-0.020	0.042	-0.450	1199	0.652
High	0.080	0.055	1.467	1199	0.142
Interaction SIxSC ➔Rumination					
Predictor: SC					
SI	Test Estimate	SE	T values	df	p
Low	0.088	0.045	1.935	1202	0.053.
Mean	0.006	0.036	0.163	1202	0.870
High	-0.076	0.043	-1.787	1202	0.074.
Predictor: SI					
SC	Test Estimate	SE	T values	df	Pr(>|t|) Sig.
Low	0.318	0.038	8.462	1202	< 2.2e-16***
Mean	0.248	0.032	7.764	1202	1.753e-14***
High	0.178	0.040	4.475	1202	8.359e-06***
Interaction SIxSCxDepression➔ Rumination					
Individuals with lower depression levels					
Predictor: SC					
SI	Test Estimate	SE	T values	df	p
Low	0.205	0.066	3.101	1199	0.002**
Mean	0.066	0.051	1.309	1199	0.191
High	-0.073	0.061	-1.194	1199	0.233
Predictor: SI					
SC	Test Estimate	SE	T values	Df	p
Low	0.326	0.053	6.171	1199	9.256e-10***
Mean	0.207	0.044	4.688	1199	3.077e-06***
High	0.089	0.058	1.532	1199	0.126
Individuals with higher depression levels					
Predictor: SC					
SI	Test Estimate	SE	T values	df	*p*
Low	-0.021	0.068	-0.317	1199	0.751
Mean	-0.046	0.052	-0.873	1199	0.383
High	-0.067	0.063	-1.100	1199	0.272
Predictor: SI					
SC	Test Estimate	SE	T values	df	*p*
Low	0.309	0.056	5.477	1199	5.275e-08***
Mean	0.288	0.047	6.110	1199	1.346e-09***
High	0.268	0.060	4.474	1199	8.398e-06***

#### Reappraisal

**Fig. 2:**
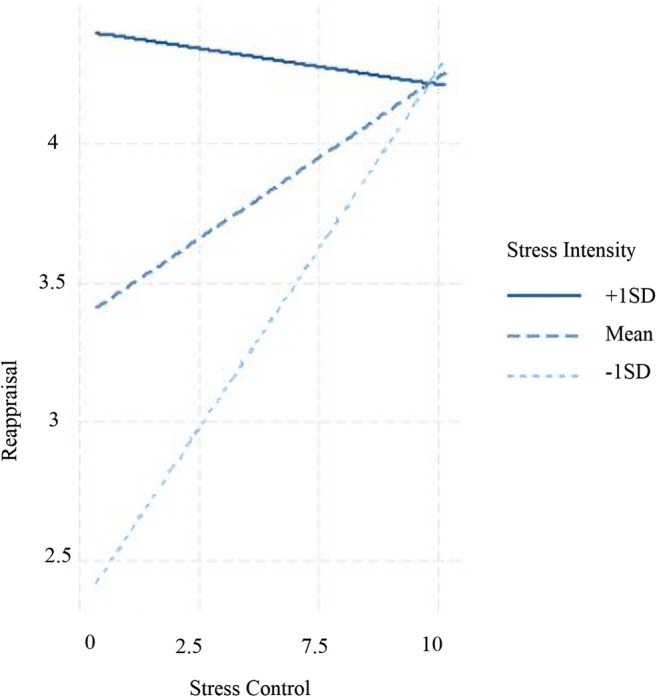
Two-way interaction between Stress Appraisals and their relation to Reappraisal use

Both higher momentary stress intensity and stress control levels were related to subsequent higher use of reappraisal. No main effects of depression or anxiety were supported. The analyses provided further support for a significant 2-way stress intensity × stress control interaction. The slopes of the interaction between stress control and reappraisal use at different stress intensity levels showed that, in situations of low-stress intensity, higher control was related to higher use of reappraisal. By contrast, no significant relations between momentary stress control and reappraisal use were supported in situations of high-stress intensity (see Fig. 2). The second set of exploratory slope analyses with stress control as a moderator did not show any specific relation between stress intensity and reappraisal use at different levels of the moderator. No significant 3-way interactions considering depression or anxiety levels as level 2 moderators were statistically significant.

### Active Coping

Higher momentary stress intensity levels were related to subsequent higher use of active coping. No other main effects of stress control, depression, or anxiety were statistically significant. The model did not provide support for 2-way interactions between stress appraisals or 3-way interactions between stress appraisals and depression or anxiety symptom levels.

#### Avoidance

**Fig. 3:**
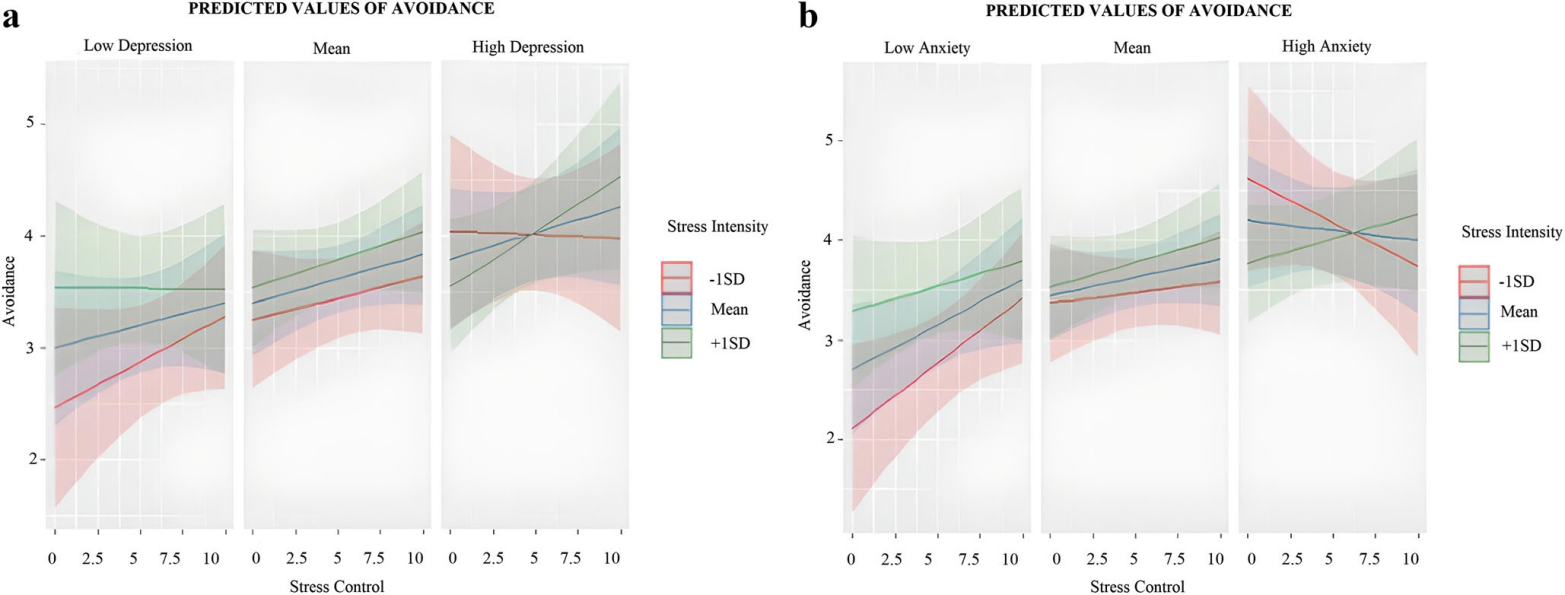
**a** Three-way interaction between Stress Appraisals and Depression and their relation to Avoidance use. **b** Three-way interaction between Stress Appraisals and Anxiety and their relation to Avoidance use

No main effects of stress intensity or stress control were supported. The main effects of depression and anxiety were both statistically significant. Higher levels of depression and anxiety were both associated with a general higher use of avoidance across the study. The 2-way stress intensity x stress control interaction was not significant, but there were statistically significant 3-way interactions between stress intensity, stress control, and both forms of affective symptomatology levels. For depression, the main slope analyses (i.e., stress intensity as a moderator) did not support any specific interaction effect in participants with low depression levels. However, for participants with high depression levels in situations of high-stress intensity, higher stress control was related to higher use of avoidance (see Fig. 3a). Exploratory slope analyses considering stress control as a moderator showed that higher stress intensity was related to higher use of avoidance for participants with low depression levels in situations of low-stress control. By contrast, for participants with high depression levels, in situations of low-stress control, higher stress intensity was related to lower use of avoidance (trend). As for anxiety, the main slope analyses (i.e., stress intensity as a moderator) showed that higher stress control was related to higher use of avoidance for participants with low anxiety levels, specifically in situations of low-stress intensity. By contrast, for participants with high anxiety levels, higher stress control was related to higher use of avoidance, specifically in situations of high-stress intensity (Fig. 3b). Exploratory slope analyses considering stress control as a moderator showed that higher stress intensity was related to higher use of avoidance for participants with low anxiety levels in situations of low-stress control. By contrast, higher stress intensity was related to lower use of avoidance for participants with high anxiety levels in situations of low-stress control.

#### Rumination

**Fig. 4:**
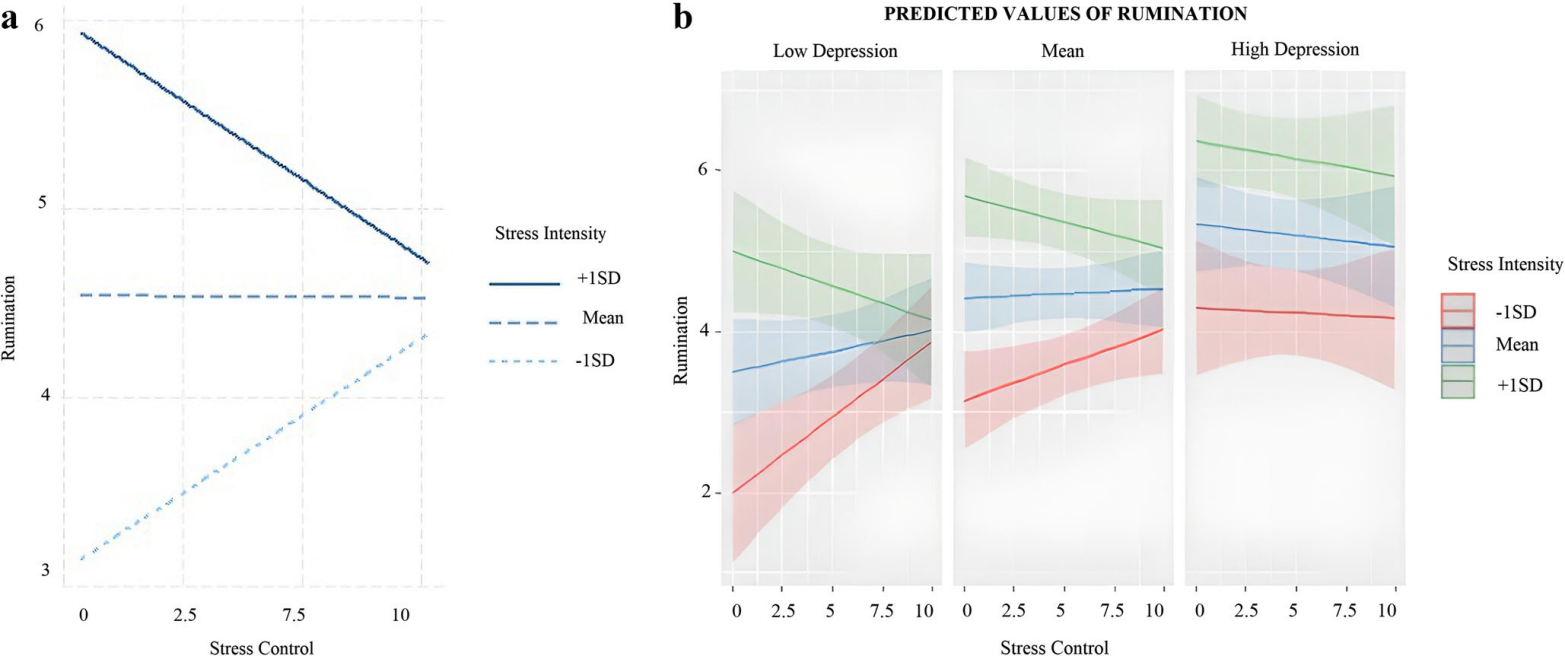
**a** Two-way interaction between Stress Appraisals and their relation to Rumination use. **b** Three-way interaction between Stress Appraisals and Depression and their relation to Rumination use

Higher use of rumination was related to higher levels of stress intensity but not to stress control. Higher individual levels of depression and anxiety were both associated with higher use of rumination across the study. The model provided support for a significant 2-way stress intensity × stress control interaction. As shown in Fig. 4a, the main slope analyses showed a trend for an inverse effect of stress control in rumination use at different stress intensity levels. This is in line with the hypothesized effects. In situations of low intensity, higher control was related to higher use of rumination (*p=* 0.05). By contrast, in situations of high intensity, higher control was associated with lower use of rumination (*p=* 0.07). A 3-way interaction between stress intensity, stress control, and depression emerged. The main slope analyses considering stress intensity as a moderator showed that the main stress control-rumination use interaction reported above was supported for participants reporting low depression levels. For participants with low levels of depression, higher stress control was significantly related to higher use of rumination in situations of low-stress intensity. This relation was not supported for participants with high depression scores. No other interaction effects emerged in exploratory analyses examining stress control as a moderator.

### Power Analyses

Post hoc power analyses were performed to determine the statistical (post hoc) power to detect the 2-way and 3-way interactions analyzed. These results showed a high statistical power for the above reported 2-way interactions (*β=* 0.864) and moderate power for the 3-way interactions (*β=* 0.714).

### Supplementary Results for Other ER Strategies Assessed in the Study

Additional analyses examined worry, mental change, external distraction, and future planning as dependent variables. Supplement 2 details all these models as well as the between- and within-person correlations between all the ER strategies and stress appraisal measures in the study.

#### Worry

Higher levels of stress intensity appraisals, but not stress control, were related to higher use of worry. Higher individual levels of depression and anxiety were both related to higher general use of worry across the study. The model provided support for a significant 2-way stress intensity × stress control interaction. The simple slopes of the interaction showed that lower stress control was related to higher use of worry in situations of high-stress intensity (see S2 Figure 1). No other interaction effects were statistically supported.

#### Mental change

Higher momentary appraisals of stress intensity and stress control were both related to higher use of mental change. Individual levels of depression and anxiety were not associated with the general use of this strategy across the study. The 2-way stress intensity × stress control interaction was marginally significant, whereas a significant 3-way stress intensity × stress control × anxiety (but not depression) was supported. For participants with low anxiety levels, specifically in high-stress intensity situations, a higher stress control was related to lower use of mental change. This specific relation was absent in participants with high levels of anxiety, who showed a general relation between higher stress control and higher use of mental change, irrespective of stress intensity levels (see Figure S2 2b).

#### External Distraction

No significant main effects or interaction effects were statistically supported for external distraction use, except a main effect of anxiety. Thus, higher individual levels of anxiety were related to higher general use of external distraction across the study.

#### Future Planning

Higher momentary appraisals of stress intensity and stress control were both related to higher use of future planning. Higher individual levels of anxiety were further related to higher general use of future planning across the study. Depression levels were not associated with the general use of this strategy and neither 2-way nor 3-way interactions were statistically supported.

## Discussion

The present study provides an investigation of how the interplay between primary (i.e., intensity) and secondary (i.e., controllability) stress appraisals is related to the flexible momentary use of different ER strategies in stressful situations in daily life. Results showed that, higher controllability was related to higher use of reappraisal in situations of low-stress intensity. This relation is in line with the proposal that reappraisal is a preferred strategy in situations of low intensity (Sheppes et al., 2014), but does not support the hypothesis that it would be preferentially used in situations of lower perceived controllability (e.g., Sheppes et al., 2009; Haines et al., 2016; Troy et al., 2013). Interestingly, this was also found for rumination (i.e., higher use in situations of low-stress intensity and high-stress controllability). These observations suggest that these two ER strategies might serve similar regulatory purposes, and plausibly be adaptive when used in specific situations. It is possible that dwelling on feelings and problems as well as looking at things from a different perspective might aid the regulation of low-intensity emotions that are appraised as controllable. However, other strategies such as active coping might be preferable in controllable situations when demands and resulting emotions are more intense (Troy, 2015). Regarding rumination, it was also found that higher levels of controllability were related to lower use of rumination in high-stress intensity situations. This shows how controllability appraisals may help to reduce rumination use (Kircanski et al., 2015), specifically in high-stress intensity situations that may require other forms of ER such as distraction (Sheppes et al., 2011, 2014) or active coping (David & Suls, 1999).

Nonetheless, the 2-way interactions between stress intensity and controllability were not significant for the use of neither active coping nor mental avoidance. These results differ from previous research showing that frequent use of avoidance and active coping in response to stressors is characterized by lower and higher controllability, respectively (David & Suls, 1999). The null finding for active coping is particularly relevant based on contemporary conceptualizations of its functionality to cope with stressors that are appraised as controllable (Troy, 2015). One potential explanation for this null finding is that the item used to assess active coping was too broad and rather reflected situational outcomes (see Haines et al., 2016, for similar findings). More fine-grained assessments of the use of different active coping strategies may be more informative in future research.

As for avoidance and rumination, results in our study showed that individual levels of depression and anxiety symptoms modulated the interplay between stress appraisals to influence their momentary use. This is consistent with recent ESM findings by Goodman et al. (2021) who found higher use of disengagement strategies including rumination, and mental avoidance as a function of social anxiety, but no impact of anxiety status on the use of engagement strategies including reappraisal, and problem-solving. In our study, for participants with low anxiety levels, higher stress controllability was related to higher use of avoidance in low-stress intensity situations. For participants with high depression or anxiety levels, higher stress controllability was related to higher use of avoidance specifically in high-stress intensity situations. Furthermore, participants reporting high levels of anxiety also used more avoidance in situations appraised as low in intensity and uncontrollable. As such, these findings differ from the results for reappraisal and rumination (i.e., higher use in situations low in stress-intensity but high in stress-controllability). Ultimately, results suggest that particularly anxiety might affect the appropriate use of avoidance strategies during daily life functioning. By contrast, the use of rumination was specifically impacted by depression. Results showed that the general flexible use of stress control-based rumination at different intensity levels, as mentioned above, was specific for participants reporting low depression levels. By contrast, participants reporting higher depression levels reported more frequent rumination use, irrespective of controllability appraisals, supporting an inflexible use of rumination across contexts (Nolen-Hoeksema et al., 2008).

Despite the novelty of these findings in research on ER flexibility, some limitations should be noted. First, the study was conducted with an undergraduate sample characterized by a broad range of subclinical symptom levels. It is thus unclear to what extent the present findings can generalize to samples diagnosed with clinical affective disorders. Second, further research should also consider the directionality of the interplay between ER flexibility and affective symptomatology. In the present study, we tested the impact of current depression and anxiety levels on the relations between stress appraisals and ER strategies. However, inflexible ER is thought to further account for future affective disturbances. Thus, further research should also consider the role of inflexible ER strategies use in predicting future affective symptomatology increases. Third, the present study considers ER use in the context of stressful situations in daily life. Thus, the results cannot be generalized to other forms of emotion-eliciting contexts that require ER. Future studies should consider the analysis of primary and secondary appraisals and their interaction when analysing how ER strategies are momentarily (and potentially flexibly) used in other contexts requiring different forms of ER (e.g., situations inducing specific negative emotions related to anger, or situations inducing positive emotions). Fourth, our ESM protocol comprised three prompts per day. It could be argued that this temporal resolution might not be sufficient to fully capture the entire unfolding of stress appraisals and ER strategy use relations. However, the chosen sampling frequency in this study was informed by previous ESM studies (Connolly & Alloy, 2017; Heiy & Cheavens, 2014). Finally, participants were predominantly female (87%) and future research should extend current findings in samples with more balanced distributions in terms of gender.

Despite these limitations, the study provides novel insights into the use of ER strategies in daily life in relation to momentary stress appraisals, and the impact of affective symptomatology on such relations. These findings have the potential to inform new innovative frameworks on ER flexibility and to improve the understanding of inflexible processes of ER that are affected by depression and anxiety.

## Supplementary Information

ESM 1 (DOCX 295 kb)

## Footnotes

1 The current study was part of a larger ESM project monitoring fluctuations and interplays among affective states and cognitive processes. Relevant variables for this study were defined and assessed as described below, whereas the full set of variables included in the project is reported in Supplement 1.

2 No time-lagged variables were used to test the hypotheses. This is because contemporary research claims that the inclusion of lagged dependent variables violates statistical assumptions of multilevel model, threatening analysis validity (McNeish & Hamaker, 2020). Nonetheless, for the sake of clarity and comparability with previous ESM research using this approach, a replication of all models with t-1 lagged dependent variables as predictors is reported in Supplement 3. Results from all these different models were similar to the ones reported using no time-lagged models in the main manuscript. Further, additional models are reported in Supplement 4, considering the influence of the factors “day” and “moment of the day” (i.e., the three daily occasions coded as “1-morning”, “2-afternoon”, and “3-evening”) to address for the potential influence of these sources of non-independence. None of the main significant effects changed as a function of the inclusion of these further variables.
