# Supplementary material for: Moment-to-Moment Interplay Among Stress Appraisals and Emotion Regulation Flexibility in Daily Life
Source: Affect Sci. 2022 Jun 6;3(3):628–40. doi: 10.1007/s42761-022-00122-9 (PMC9537410; doi:10.1007/s42761-022-00122-9)
Supplement: Supplementary file 1 — (DOCX 295 kb) [file 42761_2022_122_MOESM1_ESM.docx]

**Supplement 1: Further variables assessed in the study.**

Demographic Variables:

· Sex

· Age

Other Baseline Questionnaires:

· Cognitive Emotion Regulation Questionnaire (CERQ; Garnefski, Kraaij, & Spinhoven, 2002). This questionnaire assesses habitual use of nine different cognitive emotion regulation strategies: self-blame, acceptance, rumination, positive refocusing, refocus on planning, positive reappraisal, putting into perspective, catastrophizing and others-blame.

· Ruminative Responses Scale (RRS; Treynor, Gonzalez & Nolen-Hoeksema, 2003). This scale has two subscales: reflection and brooding. For this study we only used the five questions related to the brooding subscale.

ESM Assessment:

· Positive Emotions: cheerful, content, energetic and relaxed

· Negative Emotions: nervous, content, irritable and tired

· Emotion Regulation Strategies:

o Worry: this strategy refers to repeatedly thinking on the problems that one situation could create to the person in the future. In accordance to previous ESM research (Kircanski et al., 2015) the question used to evaluate this strategy in response to ongoing stressful experiences was “… have you been worrying about things that could happen?”

o Mental change: this item was also framed on previous ESM research monitoring different forms of momentary cognitive reappraisal use in response to stress (Haines et al., 2016) as “…have you changed the way you were thinking about the situation?”

o External distraction: this strategy reflects an external mental focus avoidance (external distraction), referred to the extent to which participants tried to distract themselves from ongoing stressful situations: “… have you tried to distract yourself from what was going on?”

o Future Planning: it was framed in accordance to problem solving models (D’Zurilla, Nezu, & Maydeu-Olivares, 2002) considering this as a form of intentional planning of future behaviours to prevent negative consequences of stress situations that are similar to the ones previously experienced: “… have you thought about what you could do to solve similar upcoming situations in the future?”

· Motivation State Questions:

o Promotion focus 1: to what extent has it been important to you achieving goals or aims that were relevant in your life?

o Promotion focus 2: to what extent have you been focused on searching ways to have fun or enjoy yourself?

o Prevention focus 1: to what extent it has been important to you solving problems or getting ready to cope with potential difficulties?

o Prevention focus 2: to what extent have you been focused on searching ways to feel secure, protected or calmed?

· Scramble Sentence Task (SST): this task evaluated cognitive interpretation biases of participants in the study at each ESM assessment. It consisted of a cognitive task in which participants must generate grammatically correct sentences with 5 out of 6 scrambled words (e.g., future dismal looks the bright very) that appeared on the mobile phone screen in a determined period of time -7 seconds-. Scrambled words could always be solved in a positive or a negative sentence (e.g., “the future looks very bright” vs. “the future looks very dismal”). People who generate positive meaning sentences more often during the task have a positive interpretation bias, which is directly related with lower levels of anxiety and depression and higher use of reappraisal. People who generate negative meaning sentences more often have a negative interpretation bias, which is related to higher levels of anxiety and depression and to a higher use of rumination (see Blanco, Boemo & Sanchez-Lopez, 2021).

· Questions about app’s usability and perceived user experience (assessed after finishing the ESM protocol):

o I think I would like to use this type of App frequently

o I found the App unnecessarily complex

o I thought the App was easy to use

o I think that I would need the support of a technical person to be able to use this App

o I found the various functions in this system were well integrated

o I thought there were too much inconsistency in this App

o I would imagine that most people would learn to use this App very quickly

o I found the App very cumbersome to use

o I felt very confident using the App

o I needed to learn a lot of things before I could get going with this App

o I would recommend to a friend or relative to use this type of App

o I felt more nervous than usual, while being vigilant to receive the App’s notifications

o The number of exercises to perform in each signal was excessive

o Using the App has helped me to be more conscious about my emotional and cognitive responses through the day and across days

o I would have liked to receive and visualize information about my emotional responses at each time I was using the App

o I would have liked to receive and visualize information about my emotional responses at the end of the study

o Score from 0 to 10 your overall satisfaction with the App

**Supplement 2: Full set of results for other ERS in the study, not reported in the main analyses**

Means, SDs, and intra-class correlation coefficients (ICCs) were estimated for use of worry, mental change, external distraction and future planning, using intercept-only models. The Supplementary 2 (i.e., S2) Table 1 presents the mean level with the 95% CI, the SDs of the within- and between-person levels, the ICC and the percentage of the total variability between and within-subject for each of these ERS variables. Table 2 shows the correlations between the ER strategies and stress appraisals, both between and within subjects.

S2 Table 1: *Mean level, SDs of the within- and between-person levels, ICC and the percentage of the total variability between and within-subject for the strategies of Worry, Mental Change, External Distraction and Future Planning.*

| Intercept-only model | Mean level Estimate  95% CI (Lower-Upper) | SD  Between subject | SD  Within-subject | ICC | % Between subjects variance | % Within-subject variance |
| --- | --- | --- | --- | --- | --- | --- |
| Worry | 4.65 (4.27-5.04) | 1.80 | 2.34 | 0.37 | 37.18% | 62.81% |
| Mental change | 3.46 (3.16-3.76) | 1.40 | 2.07 | 0.31 | 31.39% | 68.60% |
| External distraction | 4.64 (4.29-4.99) | 1.64 | 2.29 | 0.33 | 33.89% | 66.10% |
| Future Planning | 4.35 (3.93-4.76) | 1.98 | 2.16 | 0.45 | 45.58% | 54.41% |

S2 Table 2: *Correlation between ER strategies and stress appraisals.*

Correlation between subjects

|  | Stress Intensity | Stress Control | Reappraisal | Mental Change | Active Coping | Future Planning | Avoidance | Distraction | Rumination | Worry |
| --- | --- | --- | --- | --- | --- | --- | --- | --- | --- | --- |
| Stress Intensity | 1 |  |  |  |  |  |  |  |  |  |
| Stress Control | -.42** | 1 |  |  |  |  |  |  |  |  |
| Reappraisal | .30** | .07 | 1 |  |  |  | . |  |  |  |
| Mental Change | .30** | -.01 | .87** | 1 |  |  |  |  |  |  |
| Active Coping | .41** | .01 | .69** | .68** | 1 |  |  |  |  |  |
| Future Planning | .37** | .15 | .53** | .56** | .69** | 1 |  |  |  |  |
| Avoidance | .15 | -.26** | .37** | .49** | .45** | .29** | 1 |  |  |  |
| Distraction | .20* | -.21* | .43** | .50** | .55** | .40** | .69** | 1 |  |  |
| Rumination | .53** | -.21* | .46** | .46** | .47** | .64** | .28** | .46** | 1 |  |
| Worry | .72** | -.39** | .27** | .30** | .38** | .49** | .19* | .35** | .77** | 1 |

Notes. * p > .05; ** p > .01

Correlation within subjects

|  | Stress Intensity | Stress Control | Reappraisal | Mental Change | Active Coping | Future Planning | Avoidance | Distraction | Rumination | Worry |
| --- | --- | --- | --- | --- | --- | --- | --- | --- | --- | --- |
| Stress Intensity | 1 |  |  |  |  |  |  |  |  |  |
| Stress Control | -.31** | 1 |  |  |  |  |  |  |  |  |
| Reappraisal | .18** | -.01 | 1 |  |  |  | . |  |  |  |
| Mental Change | .14** | .01 | .49** | 1 |  |  |  |  |  |  |
| Active Coping | .30** | -.11** | .34** | .32** | 1 |  |  |  |  |  |
| Future Planning | .26** | -.03 | .32** | .27** | .40** | 1 |  |  |  |  |
| Avoidance | .04 | .05 | .14** | .11** | .14** | .12** | 1 |  |  |  |
| Distraction | .03 | .03 | .15** | .10** | .19** | .10** | .32** | 1 |  |  |
| Rumination | .30** | -.11** | .28** | .26** | .21** | .23** | .03 | .12** | 1 |  |
| Worry | .44** | -.17** | .31** | .25** | .33** | .32** | .05* | .10** | .41** | 1 |

Notes. * p > .05; ** p > .01

**ERS changes as a function of situational fitted stress intensity and control appraisals**

All models are reported in S2 Table 3.

S2 Table 3

|  | Fixed effects | | | | |
| --- | --- | --- | --- | --- | --- |
| Model | Effect | Estimate. | *SE* | *t* | *p* |
| Worry at t | Intercept  Stress Intensity at t  Stress Control at t  Depression at t  Stress Intensity x Stress Control  Stress Intensity x Depression  Stress Control x Depression  Stress Intensity x Stress Control x Depression | 4.617  0.365  -0.038  0.244  -0.026  0.004  0.004  0.001 | 0.169  0.030  0.033  0.042  0.009  0.007  0.009  0.002 | 27.286  12.126  -1.156  5.745  -2.802  0.546  0.463  0.580 | <0.001  <0.001  0.247  <0.001  0.005  0.585  0.643  0.561 |
|  | Anxiety at t  Stress Intensity x Stress Control  Stress Intensity x Anxiety  Stress Control x Anxiety  Stress Intensity x Stress Control x Anxiety | 0.365  -0.024  -0.001  -0.005  0.005 | 0.034  0.009  0.008  0.009  0.003 | 10.511  -2.647  -0.026  -0.634  1.620 | <0.001  0.008  0.978  0.525  0.105 |
| Mental change at t | Intercept  Stress Intensity at t  Stress Control at t  Depression at t  Stress Intensity x Stress Control  Stress Intensity x Depression  Stress Control x Depression  Stress Intensity x Stress Control x Depression | 3.438  0.109  0.069  0.007  -0.016  0.002  0.022  0.001 | 0.156  0.027  0.032  0.039  0.009  0.007  0.008  0.002 | 21.970  3.932  2.121  0.196  -1.775  0.335  2.483  0.348 | <0.001  <0.001  0.034  0.844  0.076  0.737  0.013  0.727 |
|  | Anxiety at t  Stress Intensity x Stress Control  Stress Intensity x Anxiety  Stress Control x Anxiety  Stress Intensity x Stress Control x Anxiety | 0.038  -0.016  0.001  0.024  0.007 | 0.040  0.009  0.007  0.008  0.003 | 0.957  -1.821  0.104  2.713  2.339 | 0.340  0.069  0.916  0.006  0.019 |

S2 Table 3 *(cont.)*

|  | Fixed effects | | | | |
| --- | --- | --- | --- | --- | --- |
| Model | Effect | Estimate. | *SE* | *t* | *p* |
| External distraction at t | Intercept  Stress Intensity at t  Stress Control at t  Depression at t  Stress Intensity x Stress Control  Stress Intensity x Depression  Stress Control x Depression  Stress Intensity x Stress Control x Depression | 4.668  0.035  0.044  0.079  0.016  -0.013  -0.004  -0.002 | 0.176  0.031  .039  0.044  0.010  .008  0.010  0.003 | 26.430  1.106  1.107  1.777  1526  -1.651  -0.418  -0.768 | <0.001  0.268  0.268  0.078  0.127  0.098  0.675  0.442 |
|  | Anxiety at t  Stress Intensity x Stress Control  Stress Intensity x Anxiety  Stress Control x Anxiety  Stress Intensity x Stress Control x Anxiety | 0.095  0.016  -0.003  0.002  -0.001 | 0.045  0.010  0.008  0.101  0.003 | 2.121  1.529  -0.445  0.193  -0.029 | 0.036  0.126  0.656  0.846  0.976 |
| Future planning at t | Intercept  Stress Intensity at t  Stress Control at t  Depression at t  Stress Intensity x Stress Control  Stress Intensity x Depression  Stress Control x Depression  Stress Intensity x Stress Control x Depression | 4.297  0.227  0.094  0.026  -0.012  0.006  0.008  -0.004 | 0.215  0.035  0.031  0.054  0.010  0.009  0.008  0.003 | 19.935  6.343  3.001  0.484  -1.217  0.638  0.979  -1.362 | <0.001  <0.001  0.002  0.629  0.223  0.523  0.327  0.173 |
|  | Anxiety at t  Stress Intensity x Stress Control  Stress Intensity x Anxiety  Stress Control x Anxiety  Stress Intensity x Stress Control x Anxiety | 0.149  -0.014  0.015  0.005  -0.004 | 0.053  0.010  0.009  0.008  0.003 | 2.796  -1.338  1.617  0.581  -1.268 | 0.006  0.181  0.106  0.561  0.204 |

S2 Table 4: *Summary of simple slopes analyses*

**Interaction SIxSC 🡪 Worry**

Predictor: SC

| **SI** | **Test Estimate** | **SE** | **T values** | **df** | **p** |
| --- | --- | --- | --- | --- | --- |
| Low | 0.026 | 0.042 | 0.626 | 1202 | 0.531 |
| Mean | -0.041 | 0.033 | -1.211 | 1202 | 0.226 |
| High | -0.011 | 0.039 | -2.722 | 1202 | 0.006** |

Predictor: SI

| **SC** | **Test Estimate** | **SE** | **T values** | **Df** | **p** |
| --- | --- | --- | --- | --- | --- |
| Low | 0.420 | 0.035 | 11.913 | 1202 | 2.2e-16*** |
| Mean | 0.362 | 0.030 | 11.981 | 1202 | 2.2e-16*** |
| High | 0.304 | 0.037 | 8.071 | 1202 | 1.68e-15*** |

**Interaction SIxSC 🡪 Mental Change**

Predictor: SC

| **SI** | **Test Estimate** | **SE** | **T values** | **df** | **p** |
| --- | --- | --- | --- | --- | --- |
| Low | 0.106 | 0.042 | 2.487 | 1202 | 0.013* |
| Mean | 0.065 | 0.034 | 1.901 | 1202 | 0.057. |
| High | 0.023 | 0.039 | 0.599 | 1202 | 0.549 |

Predictor: SI

| **SC** | **Test Estimate** | **SE** | **T values** | **Df** | **P** |
| --- | --- | --- | --- | --- | --- |
| Low | 0.141 | 0.033 | 4.274 | 1202 | 2.071e-05*** |
| Mean | 0.106 | 0.028 | 3.790 | 1202 | 0.001*** |
| High | 0.071 | 0.035 | 2.000 | 1202 | 0.045* |

**Interaction SIxSCxAnxiety 🡪 Mental Change**

Individuals with lower anxiety levels

Predictor: SC

| **SI** | **Test Estimate** | **SE** | **T values** | **Df** | **p** |
| --- | --- | --- | --- | --- | --- |
| Low | 0.085 | 0.059 | 1.446 | 1199 | 0.148 |
| Mean | -0.024 | 0.045 | -0.532 | 1199 | 0.594 |
| High | -0.134 | 0.056 | -2.372 | 1199 | 0.017* |

Predictor: SI

| **SC** | **Test Estimate** | **SE** | **T values** | **df** | **p** |
| --- | --- | --- | --- | --- | --- |
| Low | 0.200 | 0.048 | 4.139 | 1199 | 3.725e-05*** |
| Mean | 0.106 | 0.039 | 2.701 | 1199 | 0.006** |
| High | 0.012 | 0.051 | 0.232 | 1199 | 0.816 |

Individuals with higher anxiety levels

Predictor: SC

| **SI** | **Test Estimate** | **SE** | **T values** | **df** | **p** |
| --- | --- | --- | --- | --- | --- |
| Low | 0.137 | 0.064 | 2.132 | 1199 | 0.033* |
| Mean | 0.164 | 0.048 | 3.353 | 1199 | 0.001*** |
| High | 0.190 | 0.059 | 3.212 | 1199 | 0.001** |

Predictor: SI

| **SC** | **Test Estimate** | **SE** | **T values** | **df** | **P** |
| --- | --- | --- | --- | --- | --- |
| Low | 0.089 | 0.052 | 1.721 | 1199 | 0.085. |
| Mean | 0.112 | 0.041 | 2.720 | 1199 | 0.006** |
| High | 0.135 | 0.053 | 2.540 | 1199 | 0.011* |

**Worry.** The use of worry was associated to higher appraisals of stress intensity. Also, higher levels of depression and anxiety were both related to a higher use of worry across the study. The model also suported a significant Stress Intensity x Stress Control interaction. The simple slopes of the interaction showed that, at situations of high Stress Intensity, a higher appraisal of Stress Control was related to a lower use of worry (see S2 Figure 1).

S2 Figure 1. *Two-way interaction between Stress Appraisals and its relation to subsequent Worry use.*

**
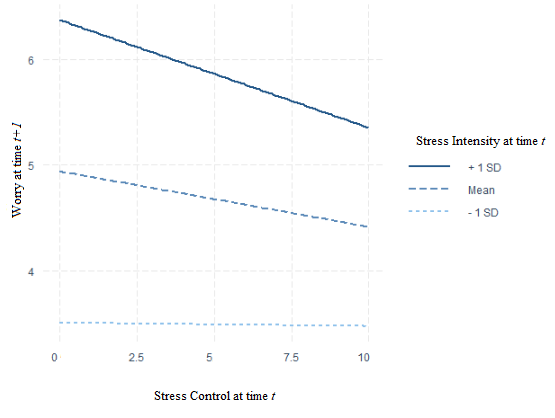
**

**Mental change.** The use of this strategy was associated with higher appraisals of stress intensity and controllability. The Stress Intensity x Stress Control interaction was also significant. The slopes of the interaction between Stress Control appraisals and Mental Change changes at different +1SD, average and -1SD Stress Intensity levels showed that, at situations of low Stress Intensity, a higher appraisal of Stress Control was related to a higher use of mental change (see S2 Figure 2a).

This model also supported a significant three-way stress intensity x stress control x anxiety level interaction. Participants with lower levels of anxiety used this strategy to a greater extent in stressful situations appraised as high in intensity and low in controllability, and to a lesser extent when appraised the situation as highly intense and highly controllable. In contrast, participants with higher levels of anxiety used mental change to a greater extent when they appraised situations as more controllable, regardless their appraisals of stress intensity (see Figure S2 2b).

| S2 Figure 2a. *Two-way interaction between Stress Appraisals and it relation to subsequent Mental Change use.* | S2 Figure 2b. *Three-way interaction between Stress Appraisals and Anxiety and it relation to subsequent Mental Change use.* |
| --- | --- |
| 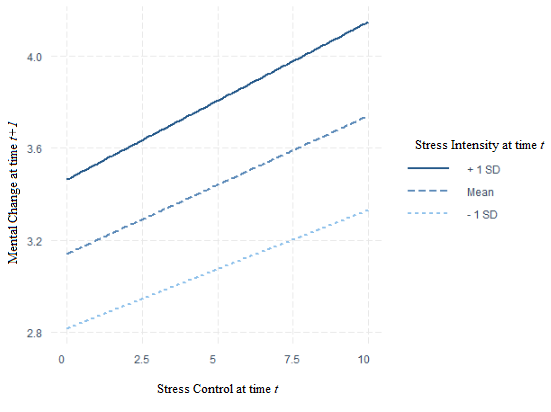 | 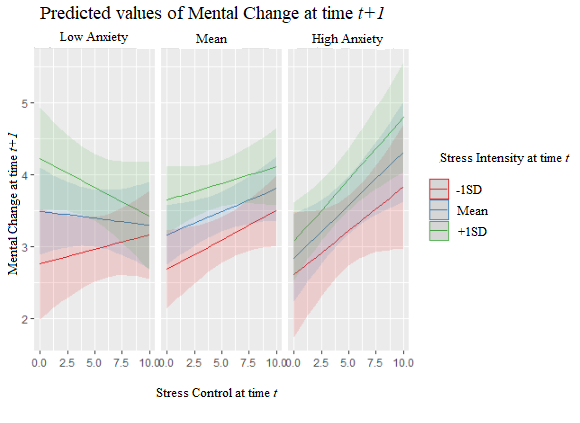 |

**External distraction.** Higher levels of anxiety were related to a higher use of distraction across the study. This model neither showed significant 2-way interactions between stress appraisals, nor 3-way interactions between stress appraisals and depression or anxiety level to account for momentary use of external distraction.

**Future Planning.** A higher use of future planning was related to higher appraisals of stress intensity and controllability. Also, higher levels of anxiety were associated with a higher use of Future Planning across the study. The model neither supported significant 2-way or 3-way interactions between stress appraisals and depression or anxiety levels to account for momentary use of future planning.

**Supplement 3: Time-lagged models for each ERS variable in the study**

S3 Table 1. *Results considering time-lagged predictors for each ERS reported in the main manuscript.*

|  | Fixed effects | | | | |
| --- | --- | --- | --- | --- | --- |
| Model | Effect | Estimate. | *SE* | *t* | *p* |
| Reappraisal at t | Intercept  Reappraisal at t-1  Stress Intensity at t  Stress Control at t  Depression at t  Stress Intensity x Stress Control  Stress Intensity x Depression  Stress Control x Depression  Stress Intensity x Stress Control x Depression | 3.860  -0.030  0.123  0.036  -0.0254  -0.031  -0.004  0.021  0.001 | 0.165  0.029  0.032  0.032  0.041  0.010  0.008  0.008  0.003 | 23.356  -1.015  3.788  1.236  -0.608  -2.996  -0.550  2.355  0.289 | <0.001  0.309  <0.001  0.256  0.544  0.002  0.582  0.018  0.772 |
|  | Anxiety at t  Stress Intensity x Stress Control  Stress Intensity x Anxiety  Stress Control x Anxiety  Stress Intensity x Stress Control x Anxiety | 0.016  -0.030  -0.009  0.026  0.005 | 0.042  0.010  0.008  0.008  0.003 | 0.402  -2.923  -1.096  2.998  1.727 | 0.688  0.003  0.273  0.002  0.084 |
| Active Coping at t | Depression at t  Stress Intensity x Stress Control  Stress Intensity x Depression  Stress Control x Depression  Stress Intensity x Stress Control x Depression | -0.001  -0.007  -0.016  0.011  -0.003 | 0.042  0.010  0.009  0.009  0.003 | -0.005  -0.728  -1.648  1.211  -0.985 | 0.995  0.466  0.099  0.226  0.324 |
|  | Anxiety at t  Stress Intensity x Stress Control  Stress Intensity x Anxiety  Stress Control x Anxiety  Stress Intensity x Stress Control x Anxiety | 0.082  -0.008  0.004  0.017  0.002 | 0.042  0.010  0.010  0.009  0.003 | 1.969  -0.764  0.406  1.945  0.768 | 0.051  0.445  0.684  0.052  0.442 |

S3 Table 1. *Results considering time-lagged predictors for each ERS reported in the main manuscript (cont.)*

|  | Fixed effects | | | | |
| --- | --- | --- | --- | --- | --- |
| Model | Effect | Estimate. | *SE* | *t* | *p* |
| Avoidance at t | Intercept  Avoidance at t-1  Stress Intensity at t  Stress Control at t  Depression at t  Stress Intensity x Stress Control  Stress Intensity x Depression  Stress Control x Depression  Stress Intensity x Stress Control x Depression | 3.569  0.048  0.060  0.051  0.089  0.012  -0.015  0.007  0.007 | 0.162  0.030  0.029  0.038  0.041  0.010  0.007  0.010  0.003 | 21.945  1.587  2.060  1.346  2.184  1.185  -1.923  0.770  2.202 | <0.001  0.112  0.039  .178  0.031  0.236  0.054  0.441  0.027 |
|  | Anxiety at t  Stress Intensity x Stress Control  Stress Intensity x Anxiety  Stress Control x Anxiety  Stress Intensity x Stress Control x Anxiety | 0.089  0.013  -0.013  0.001  0.007 | 0.041  0.010  0.008  0.010  0.003 | 2.147  1.258  -1.664  0.106  2.231 | 0.034  0.208  0.096  0.915  0.025 |
| Rumination at t | Intercept  Rumination at t-1  Stress Intensity at t  Stress Control at t  Depression at t  Stress Intensity x Stress Control  Stress Intensity x Depression  Stress Control x Depression  Stress Intensity x Stress Control x Depression | 4.419  0.004  0.239  0.010  0.224  -0.030  0.010  -0.010  0.005 | 0.192  0.029  0.033  0.039  0.048  0.010  0.008  0.010  0.003 | 22.977  0.144  7.198  0.263  4.632  -2.858  1.189  -1.009  1.709 | <0.001  0.885  <0.001  0.792  <0.001  0.004  0.234  0.313  0.087 |
|  | Anxiety at t  Stress Intensity x Stress Control  Stress Intensity x Anxiety  Stress Control x Anxiety  Stress Intensity x Stress Control x Anxiety | 0.291  -0.029  0.006  0.006  0.001 | 0.045  0.010  0.009  0.010  0.003 | 6.418  -2.702  0.676  0.056  0.454 | <0.001  0.007  0.498  0.954  0.649 |

S3 Table 2. *Results considering time-lagged predictors for each ERS reported in the supplement.*

|  | Fixed effects | | | | |
| --- | --- | --- | --- | --- | --- |
| Model | Effect | Estimate. | *SE* | *t* | *p* |
| Worry at t | Intercept  Worry at t-1  Stress Intensity at t  Stress Control at t  Depression at t  Stress Intensity x Stress Control  Stress Intensity x Depression  Stress Control x Depression  Stress Intensity x Stress Control x Depression | 4.542  0.027  0.386  -0.036  0.237  -0.030  0.003  0.005  0.004 | 0.177  0.027  0.034  0.034  0.044  0.010  0.009  0.009  0.003 | 25.574  0.999  11.271  -1.039  5.292  -2.963  0.406  0.572  0.366 | <0.001  0.317  <0.001  0.298  <0.001  0.003  0.684  0.567  0.714 |
|  | Anxiety at t  Stress Intensity x Stress Control  Stress Intensity x Anxiety  Stress Control x Anxiety  Stress Intensity x Stress Control x Anxiety | 0.361  -0.029  -0.001  0.001  0.004 | 0.036  0.010  0.009  0.009  0.003 | 9.887  -2.846  -0.081  0.009  1.219 | <0.001  0.004  0.935  0.992  0.222 |
| Mental change at t | Intercept  Mental change at t-1  Stress Intensity at t  Stress Control at t  Depression at t  Stress Intensity x Stress Control  Stress Intensity x Depression  Stress Control x Depression  Stress Intensity x Stress Control x Depression | 3.398  -0.047  0.123  0.080  0.014  -0.014  0.004  0.031  0.001 | 0.160  0.030  0.030  0.037  0.040  0.010  0.008  0.010  0.003 | 21.197  -1.581  3.997  2.130  0.358  -1.417  0.516  3.091  0.457 | <0.001  0.114  <0.001  0.033  0.720  0.156  0.605  0.002  0.647 |
|  | Anxiety at t  Stress Intensity x Stress Control  Stress Intensity x Anxiety  Stress Control x Anxiety  Stress Intensity x Stress Control x Anxiety | 0.022  -0.014  0.001  0.020  0.006 | 0.040  0.010  0..008  0.010  0.003 | 0.820  -1.397  0.035  3.004  2.110 | 0.413  0.162  0.972  0.002  0.035 |

S3 Table 2. *Results considering time-lagged predictors for each ERS reported in the supplement (cont.)*

|  | Fixed effects | | | | |
| --- | --- | --- | --- | --- | --- |
| Model | Effect | Estimate. | *SE* | *t* | *p* |
| External distraction at t | Intercept  External distraction at t-1  Stress Intensity at t  Stress Control at t  Depression at t  Stress Intensity x Stress Control  Stress Intensity x Depression  Stress Control x Depression  Stress Intensity x Stress Control x Depression | 4.645  0.015  0.029  0.028  0.077  0.018  -0.009  -0.008  -0.005 | 0.185  0.031  0.035  0.041  0.046  0.011  0.009  0.011  0.003 | 25.107  0.549  0.874  0.747  1.923  1.512  -0.110  0.459  -0.382 | <0.001  0.582  0.382  0.455  0.057  0.130  0.911  0.645  0.702 |
|  | Anxiety at t  Stress Intensity x Stress Control  Stress Intensity x Anxiety  Stress Control x Anxiety  Stress Intensity x Stress Control x Anxiety | 0.091  0.017  -0.001  0.005  -0.001 | 0.047  0.01  0.009  0.011  0.003 | 1.923  1.512  -0.110  0.459  -0.382 | 0.057  0.130  0.911  0.645  0.702 |
| Future planning at t | Intercept  Future planning at t-1  Stress Intensity at t  Stress Control at t  Depression at t  Stress Intensity x Stress Control  Stress Intensity x Depression  Stress Control x Depression  Stress Intensity x Stress Control x Depression | 4.261  0.067  0.228  0.091  0.026  -0.013  0.006  0.008  -0.004 | 0.216  0.037  0.035  0.031  0.054  0.010  0.009  0.008  0.0032 | 19.665  1.791  6.392  2.897  0.404  -1.261  0.673  0.952  -1.329 | <0.001  0.073  <0.001  0.003  0.621  0.207  0.500  0.340  0.184 |
|  | Anxiety at t  Stress Intensity x Stress Control  Stress Intensity x Anxiety  Stress Control x Anxiety  Stress Intensity x Stress Control x Anxiety | 0.151  -0.014  0.015  0.004  -0.004 | 0.053  0.010  0.009  0.008  0.003 | 2.847  -1.382  1.602  0.565  -1.266 | 0.005  0.167  0.109  0.572  0.205 |

**Supplement 4: Models including time study variables (Day and Time of Day).**

S4 Table 1. *Results on the relations between symptom levels, stress appraisals and time variables across the study.*

| Model | Fixed Effects | Estimate. | *SE* | *t* | *p* |
| --- | --- | --- | --- | --- | --- |
| Stress Intensity at t | Intercept  Depression  Day  Time of day | 3.672  0.145  -0.019  0.064 | 0.267  0.037  0.051  0.088 | 13.765  3.915  -0.391  0.726 | <.0.001  <.0.001  .695  .467 |
|  | Intercept  Anxiety  Day  Time of day | 3.670  0.236  -0.020  0.063 | 0.256  0.031  0.051  0.088 | 14.314  7.689  -0.394  0.720 | <0.001  <0.001  0.693  0.471 |
| Stress Control at t | Intercept  Depression  Day  Time of day | 5.846  -0.216  -0.057  0.085 | 0.237  0.034  0.043  0.075 | 24.621  -6.396  -1.319  1.127 | <0.001  <0.001  0.187  0.259 |
|  | Intercept  Anxiety  Day  Time of day | 5.842  -0.200  -0.057  0.082 | 0.240  0.039  0.043  0.075 | 24.297  -5.119  -1.315  1.086 | <0.001  <0.001  0.188  0.277 |

S4 Table 2. *Results of models including time study variables.*

|  | Fixed effects | | | | |
| --- | --- | --- | --- | --- | --- |
| Model | Effect | Estimate. | *SE* | *t* | *p* |
| Stress Intensity |  |  |  |  |  |
| Reappraisal at t | Intercept  Stress Intensity at t  Stress Control at t  Depression at t  Day at t  Time of day at t  Stress Intensity x Stress Control  Stress Intensity x Depression  Stress Control x Depression  Stress Intensity x Stress Control x Depression | 4.751  0.132  0.058  -0.031  -0.112  -0.237  -0.028  -0.013  0.013  0.001 | 0.244  0.030  0.029  0.040  0.041  0.071  0.009  0.008  0.008  0.002 | 19.432  4.312  1.975  -0.787  -2.705  -3.321  -2.969  -1.628  1.660  0.165 | <0.001  <0.001  0.048  0.433  0.006  <0.001  0.003  0.103  0.097  0.868 |
|  | Anxiety at t  Day at t  Time of day at t  Stress Intensity x Stress Control  Stress Intensity x Anxiety  Stress Control x Anxiety  Stress Intensity x Stress Control x Anxiety | 0.014  -0.109  -0.241  -0.029  -0.015  0.021  0.004 | 0.040  0.041  .071  0.009  0.008  0.008  0.003 | 0.348  -2.632  -3.377  -3.014  -1.813  2.599  1.534 | 0.725  0.008  <0.001  0.002  0.070  0.009  0.125 |
| Active Coping at t | Intercept  Stress Intensity at t  Stress Control at t  Depression at t  Day at t  Time of day at t  Stress Intensity x Stress Control  Stress Intensity x Depression  Stress Control x Depression  Stress Intensity x Stress Control x Depression | 3.940  0.229  0.001  0.020  -0.075  0.063  -0.005  -0.013  0.010  -0.002 | 0.246  0.346  0.029  0.040  0.041  0.071  0.009  0.009  0.009  0.003 | 15.963  6.672  0.037  0.253  1.803  0.882  -0.595  -1.458  1,252  -0.763 | <0.001  <0.001  0.969  0.800  0.071  0.377  0.551  0.145  0.210  0.445 |
|  | Anxiety at t  Day at t  Time of day at t  Stress Intensity x Stress Control  Stress Intensity x Anxiety  Stress Control x Anxiety  Stress Intensity x Stress Control x Anxiety | 0.076  -0.071  0.061  -0.005  0.004  0.017  0.005 | 0.040  0.041  0.071  0.009  0.009  0.008  0.005 | 1.868  -1.713  0.859  -0.594  0.457  2.087  1.648 | 0.064  0.086  0.394  0.552  0.647  0.037  0.099 |

S4 Table 2. *Results of models including time study variables (cont.)*

|  | Fixed effects | | | | |
| --- | --- | --- | --- | --- | --- |
| Model | Effect | Estimate. | *SE* | *t* | *p* |
| Stress Intensity |  |  |  |  |  |
| Avoidance at t | Intercept  Stress Intensity at t  Stress Control at t  Depression at t  Day at t  Time of day at t  Stress Intensity x Stress Control  Stress Intensity x Depression  Stress Control x Depression  Stress Intensity x Stress Control x Depression | 4.032  0.046  0.63  0.103  -0.102  -0.065  0.012  -0.014  0.001  0.007 | 0.245  0.027  0.035  0.039  0.042  0.072  0.009  0.007  0.009  0.002 | 16.425  1.685  1.815  2.587  -2.416  -0.902  1.311  -1.931  0.089  2.366 | <0.001  0.091  0.069  0.011  0.015  0.367  0.190  0.053  0.928  0.018 |
|  | Anxiety at t  Day at t  Time of day at t  Stress Intensity x Stress Control  Stress Intensity x Anxiety  Stress Control x Anxiety  Stress Intensity x Stress Control x Anxiety | 0.104  -0.100  -0.067  0.013  -0.017  -0.006  0.008 | 0.040  0.042  0.072  0.009  0.007  0.009  0.003 | 2.554  -1.377  -0.930  1.391  -2.294  -0.687  2.710 | 0.012  0.017  0.352  0.164  0.021  0.492  0.006 |
| Rumination at t | Intercept  Stress Intensity at t  Stress Control at t  Depression at t  Day at t  Time of day at t  Stress Intensity x Stress Control  Stress Intensity x Depression  Stress Control x Depression  Stress Intensity x Stress Control x Depression | 5.206  0.250  0.011  0.216  -0.044  -0.275  -0.033  0.009  -0.013  0.005 | 0.266  0.030  0.034  0.046  0.042  0.072  0.010  0.008  0.009  0.005 | 19.567  7.950  0.339  4.606  -1.032  -3.781  -3.310  1.191  -1.452  1.825 | <0.001  <0.001  0.734  <0.001  0.302  <0.001  <0.001  0.233  0.146  0.068 |
|  | Anxiety at t  Day at t  Time of day at t  Stress Intensity x Stress Control  Stress Intensity x Anxiety  Stress Control x Anxiety  Stress Intensity x Stress Control x Anxiety | 0.288  -0.049  -0.276  -0.031  0.007  -0.001  0.003 | 0.043  0.042  0.073  0.010  0.008  0.009  0.003 | 6.551  -1.163  -3.787  -3.153  0.801  -0.203  0.975 | <0.001  0.248  <0.001  0.001  0.423  0.838  0.329 |

S4 Table 2. *Results of models including time study variables (cont.)*

|  | Fixed effects | | | | |
| --- | --- | --- | --- | --- | --- |
| Model | Effect | Estimate. | *SE* | *t* | *p* |
| Stress Intensity |  |  |  |  |  |
| Worry at t | Intercept  Stress Intensity at t  Stress Control at t  Depression at t  Day at t  Time of day at t  Stress Intensity x Stress Control  Stress Intensity x Depression  Stress Control x Depression  Stress Intensity x Stress Control x Depression | 5.223  0.362  -0.044  0.244  -0.179  -0.041  -0.027  0.003  0.004  0.001 | 0.250  0.029  0.033  0.042  0.041  0.071  0.009  0.007  0.009  0.002 | 2.863  12.190  -1.345  5.747  -4.221  -0.586  -1.847  0.384  0.519  0.584 | <0.001  <0.001  0.178  <0.001  <0.001  0.557  0.004  0.700  0.603  0.559 |
|  | Anxiety at t  Day at t  Time of day at t  Stress Intensity x Stress Control  Stress Intensity x Anxiety  Stress Control x Anxiety  Stress Intensity x Stress Control x Anxiety | 0.363  -0.180  -0.045  -0.025  -0.001  -0.006  0.005 | 0.034  0.041  0.070  0.009  0.008  0.009  0.003 | 10.514  -4.361  -0.634  -2.698  -0.107  -0.60  1.700 | <0.001  <0.001  0.525  0.007  0.914  0.509  0.089 |
| Mental Change at t | Intercept  Stress Intensity at t  Stress Control at t  Depression at t  Day at t  Time of day at t  Stress Intensity x Stress Control  Stress Intensity x Depression  Stress Control x Depression  Stress Intensity x Stress Control x Depression | 3.789  0.109  0.067  0.008  -0.046  -0.107  -0.016  0.002  0.022  0.001 | 0.238  0.027  0.032  .039  0.040  0.069  0.009  0.007  0.008  0.002 | 15.872  3.927  2.060  0.211  -1.151  -1.555  -1.786  0.282  2.468  0.336 | <0.001  <0.001  0.039  0.833  0.249  0.120  0.074  0.777  0.013  0.336 |
|  | Anxiety at t  Day at t  Time of day at t  Stress Intensity x Stress Control  Stress Intensity x Anxiety  Stress Control x Anxiety  Stress Intensity x Stress Control x Anxiety | 0.038  -0.044  -0.110  -0.016  .001  0.023  0.007 | 0.040  0.040  0.069  0.009  0.007  0.008  0.003 | 0.968  -1.105  -1.601  -1.834  0.089  1.675  2.370 | 0.335  0.269  0.109  0.066  0.928  0.007  0.017 |

S4 Table 2. *Results of models including time study variables (cont.)*

|  | Fixed effects | | | | |
| --- | --- | --- | --- | --- | --- |
| Model | Effect | Estimate. | *SE* | *t* | *p* |
| Stress Intensity |  |  |  |  |  |
| External distraction at t | Intercept  Stress Intensity at t  Stress Control at t  Depression at t  Day at t  Time of day at t  Stress Intensity x Stress Control  Stress Intensity x Depression  Stress Control x Depression  Stress Intensity x Stress Control x Depression | 5.123  0.032  0.038  0.078  -0.158  0.003  0.016  -0.015  -0.003  -0.002 | 0.267  0.031  0.039  0.044  .045  0.077  0.010  0.008  0.010  0.003 | 19.151  1.030  0.969  1.780  -3.502  0.050  1.516  -1.830  -0.376  -0.754 | <0.001  0.303  0.332  0.078  <0.001  0.959  .129  0.067  0.706  0.450 |
|  | Anxiety at t  Day at t  Time of day at t  Stress Intensity x Stress Control  Stress Intensity x Anxiety  Stress Control x Anxiety  Stress Intensity x Stress Control x Anxiety | 0.095  -0.155  0.004  0.016  -0.004  0.001  0.001 | 0.45  0.045  0.077  0.010  0.008  0.010  0.003 | 2.124  -3.426  0.063  1.522  -0.528  0.157  0.024 | 0.036  <0.001  0.949  0.128  0.597  0.875  0.987 |
| Future Planning at t | Intercept  Stress Intensity at t  Stress Control at t  Depression at t  Day at t  Time of day at t  Stress Intensity x Stress Control  Stress Intensity x Depression  Stress Control x Depression  Stress Intensity x Stress Control x Depression | 4.494  0.229  0.062  0.030  -0.008  -0.065  -0.008  0.001  0.007  -0.003 | 0.280  0.029  0.030  0.053  0.041  0.071  0.009  0.007  0.008  0.002 | 16.009  7.852  2.075  0.573  -0.212  -0.927  -0.858  0.039  0.890  -1.103 | <0.001  <0.001  0.038  0.567  0.831  0.354  0.391  0.968  0.373  0.270 |
|  | Anxiety at t  Day at t  Time of day at t  Stress Intensity x Stress Control  Stress Intensity x Anxiety  Stress Control x Anxiety  Stress Intensity x Stress Control x Anxiety | 0.154  -0.006  -0.065  -0.008  0.10  0.005  -0.002 | 0.052  0.041  0.071  0.009  0.007  0.008  0.003 | 2.968  -0.157  -0.926  -0.904  1.304  0.598  -0.853 | 0.003  0.874  0.354  0.365  0.193  0.549  0.393 |
